# Amino acids: Missing link in preeclampsia pathogenesis?

**DOI:** 10.1007/s00394-026-03977-x

**Published:** 2026-05-14

**Authors:** Iclal Sena Gezer, Hasan Altinsoy, Ayse Gulcin Bastemur, Ozlem Dogan, Atakan Tanacan, Fatma Doga Ocal, Dilek Sahin, Nuray Yazihan

**Affiliations:** 1 Interdisciplinary Food, Metabolism and Clinical Nutrition Department, Institute of Health Sciences, Ankara University, Ankara, Turkey; 2 Internal Medicine Division, Department of Pathophysiology, Faculty of Medicine, Ankara University, Morfoloji Building, Sihhiye, Ankara, Turkey; 3 Division of Perinatology, Department of Obstetrics and Gynecology, Ministry of Health, Ankara City Hospital, University of Health Sciences, Ankara, Turkey; 4 Department of Medical Biochemistry, Faculty of Medicine, Ankara University, Ankara, Turkey

**Keywords:** Preeclampsia, Diet, Serum, AAs, Arginine, BCAA

## Abstract

**Purpose:**

This study was designed to determine maternal dietary and serum amino acid (AA) levels as risk factors for preeclampsia (PE), aiming to identify the potential of specific AAs as biomarkers that could lead to novel dietary intervention strategies.

**Methods:**

This prospective observational study included a total of 84 pregnant women (27 with PE and 57 controls). Maternal serum AA profiles were quantified using liquid chromatography-mass spectrometry (LC–MS), and dietary AA intake was assessed via three-day dietary recalls, usual intake was estimated using the Multiple Source Method (MSM) to correct for within-person variability. The potential of AA as a risk factor was evaluated using the Receiver Operating Characteristic (ROC) curve analysis, and key mechanisms involved in AA metabolism were identified through pathway analysis.

**Results:**

Dietary assessment uncovered a altered dietary amino acid pattern (AUC > 0.8), characterized by low intake of arginine, alanine, and glycine, glutamic acid, histidine, proline, serine, threonine, and tryptophan, alongside high total branched-chain amino acid (BCAA) and leucine consumption. Although dietary total protein intake was lower in PE, a higher intake of animal protein was observed. Concurrently, serum glutamine, asparagine, phenylalanine, 3-methylhistidine, cysteine, threonine, valine, BCAA, EAA, and aromatic AAs (AUC > 0.8) in serum were found to be potential risk factors for PE. Our results establish that dietary AA patterns and serum AA changes are independent, key risk factors, together providing a more comprehensive etiological model of PE.

**Conclusion:**

This study showed that a dietary imbalance in specific AAs represents a modifiable risk factor for PE. This finding, alongside an altered serum AA profile, opens new avenues for targeted nutritional interventions in high-risk pregnancies.

**Supplementary Information:**

The online version contains supplementary material available at 10.1007/s00394-026-03977-x.

## Introduction

Hypertensive disorders in pregnancy account for 14% and are the second leading cause of maternal deaths worldwide [1]. Preeclampsia (PE) is diagnosed after 20 weeks of gestation when new hypertension occurs with either proteinuria or end-organ dysfunction [2]. Women who develop PE have a higher risk for cardiovascular disease and diabetes, and reduced life expectancy, while infants from PE pregnancies are at greater risk for early birth, perinatal death, and future cardiovascular or metabolic disease [3, 4]. Despite numerous established risk factors for PE, preventive treatment and prediction options are limited [5].

AAs are protein components that also serve as precursors in cell signaling pathways, metabolism, gene regulation, neurotransmission, the creation of hormones, coenzymes, nucleic acids, and have immunomodulatory functions in biochemical pathways; involved in the production of nitric oxide, histamine, antioxidants, and hydrogen peroxide [6].

The relationship between dietary AA intake and blood pressure has recently gained considerable attention. Although the dietary AA profile has not been evaluated in PE, studies have found the dietary AA profile to be associated with hypertension [7–10]. A previous study by Ma et al. [11] evaluated dietary methionine concentrations in PE. Higher methionine intake was associated with an increased risk of PE.

As AAs taken through diet interact with each other, taking several AAs at the same time may alter the risk of hypertension [12]. The potential for these AA profiles, which vary in metabolism, to be used as biomarkers has gained importance in recent years [13]. The dietary component analysis approach is a method that considers the cumulative and interactive effects between food components and reflects the complexity of human nutrition [12, 14].

To the best of our knowledge, no research has investigated dietary AA components in PE. While changes in serum AA profiles in PE have been investigated in numerous studies [15–17], the role AAs play in PE has not been fully understood.

With this aim, this study was designed to determine maternal dietary and serum AA levels as risk factors for PE, aiming to identify the potential of specific AAs as biomarkers that could lead to novel dietary intervention strategies.

## Materials and methods

### Patient population

The present case–control study included pregnant women aged 18–45 in their third trimester (28—40 weeks) diagnosed with PE (n = 27), and normotensive (n = 57), admitted to the Department of Perinatology, Turkish Ministry of Health, Ankara City Hospital between 25 November 2023 and 10 June 2024. The normotensive controls were randomly selected during the identical study period, ensuring they represented a comparable source population. PE patients were diagnosed based on the criteria of the American College of Obstetricians and Gynecologists (ACOG) [2] at the Ankara Bilkent City Hospital Perinatology Clinic. Pregnant women with multiple pregnancies, diabetes mellitus, membrane ruptures, infection, chronic autoimmune diseases, fetal anomalies, stillbirth, maternal renal disorders, pre-existing medical conditions that could impact AA levels, use of medications affecting AA levels, or other significant medical conditions were not included in the study. Active smoking and alcohol consumption were not included because of their inflammatory and oxidative effects.

The study adheres to the Declaration of Helsinki, and ethical approval for the study has been obtained from the institutional clinical research ethics committee (Ethical Number: TABED-2-24-674). All participants provided written informed consent before enrolment. Consent documents included assurances that biological samples would be securely stored and used for research purposes. The sample size was calculated using G*Power (v3.1.9.7) based on serum glycine concentrations reported by López-Quesada et al. (2003). To detect an expected effect size (Cohen's *d*) of 1.31 using a two-tailed independent t-test with 95% power and 0.05 error size, a minimum of 17 participants per group (total *n* = 34) was required. Our final sample of 27 preeclampsia patients and 57 controls adequately meets this requirement.

### Dietary assessment and AA intakes

The dietary recalls were obtained simultaneously with the blood sampling at the time of hospital admission. This timing ensured that the collected data reflected the patients' routine, habitual dietary intake before the initiation of any hospital-prescribed diet programs or medical interventions. Dietary assessment was performed via 24-h dietary recalls administered for three days (two weekdays and one weekend day) by an experienced dietitian (I.S.G.). Food consumption data were collected using household measures to ensure accurate estimation of mean daily nutrient intakes. A photographic atlas was used to measure the portion sizes of foods [18]. Dietary AA intake was calculated using a digital nutrition database tool (Ebispro for Windows, Stuttgart, Germany; Turkish Version (BeBiS9) Istanbul program incorporates data from Bundeslebensmittelschlussel (BLS) 3.01B and USDA-SR). To estimate the usual daily intake of nutrients and correct for within-person variability (day-to-day variation), the Multiple Source Method (MSM) was employed. The MSM is a statistical method characterized by a two-part shrinkage technique applied to the residuals of two regression models: one for the probability of consumption and one for the positive daily intake data. This method allows for the estimation of individual usual intake from repeated 24-h recall data by removing the variance explained by measurement error and day-to-day fluctuations. The analyses were performed using the web-based MSM interface (Version 1.0.2e) developed by the Department of Epidemiology at the German Institute of Human Nutrition Potsdam-Rehbrücke (DIfE) (available at https://msm.dife.de/).

### LC–MS/MS analysis of serum-free AAs

Blood samples were collected from patients and centrifuged at 3000 rpm before initiating any clinical treatment, including antihypertensives, corticosteroids, or magnesium sulfate etc. were centrifuged at 3000 rpm. The supernatants were used for evaluation. In quantitative AA analysis, the sample and stable isotope-labeled internal standards were combined in an Eppendorf tube and vortexed for 30 s. After 5 min at 2–8 °C, it was centrifuged at 10,000 rpm for 5 min. The resulting supernatant was transferred to a plate and dried at 40 °C in nitrogen for 30 min. Following the drying procedure, pipetting was used to derivatize Amino Acids and Acylcarnitines by RECIPE (München/Germany) kit reagent B which is performed to facilitate comprehensive multi-amino acid profiling. It was wrapped in aluminum foil and incubated at 60 °C for 15 min. After incubation, it was dried with nitrogen for 5 min. Amino Acids and Acylcarnitines by RECIPE (München/Germany) kit reagent C (solvent) was used. The item was wrapped in aluminum foil and shaken at 250 rpm for one minute. The ready-for-injection sample was put into the insert vial. Chromatographic separation was performed using a HILIC column, with mobile phases, gradient, and run time applied according to the manufacturer's validated protocol. After adjusting the Thermo TSQ Quantis LC–MS/MS instrument-equipped with a heated electrospray ionization (HESI) source, 10 µL of supernatant was injected [19–25]. Detection was achieved using multiple reaction monitoring (MRM). All measured AAs are in the L form, except for aminobutyric acid, which is in the D,L form.

Given the multiplex nature of the assay, the limits of detection (LOD) and quantification (LOQ) vary specifically for each individual amino acid and LOD and LOQ of analyzed amino acids were defined according to the manufacturer’s validation data provided with the kit. The LOD was determined as the lowest analyte concentration yielding a signal-to-noise ratio (S/N) ≥ 3, whereas the LOQ was defined as the lowest concentration that could be quantified with acceptable precision and accuracy (S/N ≥ 10). Due to the use of isotope-labeled internal standards and MRM-based detection, the method provides high analytical sensitivity, enabling reliable quantification of amino acids at low micromolar concentrations in plasma samples. Analytical performance characteristics, including analyte-specific LOD and LOQ values for individual analytes, were verified to be within the ranges specified by the manufacturer.

### Data analysis

The G Power analysis program was used to calculate the sample size. Statistical analyses were done with IBM SPSS version 26.0 software (IBM, Armonk, NY, USA). Demographic characteristics of individuals, including gestational week, birth weight, dietary and serum AA levels, were compared between groups. Data distributions were assessed with Kolmogorov–Smirnov and Shapiro–Wilk tests.

The normality of continuous variables was evaluated using the Shapiro–Wilk test/Kolmogorov–Smirnov test. Normally distributed data are presented as mean ± standard deviation (SD) and were compared between the preeclampsia and control groups using the Independent Samples t-test. Non-normally distributed variables are expressed as median (interquartile range, IQR) and were compared using the Mann–Whitney U test. To evaluate the independent associations between demographic/nutritional variables and the risk of preeclampsia while controlling for potential confounders, a multivariable logistic regression model was constructed. The model included preeclampsia as the dependent variable. Based on clinical relevance, maternal age, body mass index (BMI), parity, gestational age, total dietary protein intake, and animal protein intake were entered into the model as covariates. The Enter method was used, and model fit was assessed using the Hosmer–Lemeshow test. Adjusted Odds Ratios (aOR) and 95% Confidence Intervals (CI) were calculated. The Receiver Operating Characteristic (ROC) curve analyses were applied to assess the diagnostic performance of each AA levels, and the Youden index (J) was used to determine the optimal cut-off points of each AAs. A two-tailed P value of less than 0.05 was considered statistically significant.

Pathway analyses were performed in MetaboAnalyst 6.0 (https://new.metaboanalyst.ca/MetaboAnalyst/). The Kyoto Encyclopedia of Genes and Genomes (KEGG) database was utilized as the reference library. The analysis incorporated Over-Representation Analysis (ORA) via the hypergeometric test to evaluate pathway enrichment, and Pathway Topology Analysis via relative-betweenness centrality to determine pathway impact scores. To account for multiple testing, p-values were confirmed by using the False Discovery Rate (FDR) based on the Benjamini–Hochberg procedure.

## Results

The demographic data and clinical characteristics of cases are summarized in Table 1. PE patients had lower total protein but higher animal protein intake (*p* < 0.05). The dietary intake of AAs, alanine, arginine, glutamic acid, glycine, histidine, lysine, methionine, proline, serine, threonine, tryptophan was significantly lower in PE, whereas intake of isoleucine, leucine, valine was higher in PE (*p* < 0.05). Furthermore, subgroup analysis revealed that dietary arginine/proline ratios, intake of essential AAs (EAA), non-essential AAs (NEAA), and sulfur AAs were significantly lower, whereas intake of total branched-chain amino acids (BCAA) was significantly higher in PE (*p* < 0.05). (Table 2).

**Table 1:** Comparative analysis of mothers’ characteristics and perinatal outcomes of the groups

Variables	Preeclampsia (n = 27)	Control (n = 57)	*P* value
Maternal age (years) (IQR)	30.17 (11)	28.05 (9)	0.140
Gravida (median, IQR)	2.13 (3)	2.18 (2)	0.624
Parity (median, IQR)	0.78 (2)	1.12 (2)	0.145
Abortus (median, IQR)	0.30 (1)	0.39 (1)	0.863
Pre-pregnancy BMI (kg/m^2^)	28.84 (5.82)	24.69 (6.2)	< 0.001***
Prior to delivery BMI (kg/m^2^)	33.04 ± 5.10	29.07 ± 4.59	0.002***
Gestational week (median,IQR)	35.09 ± 2.93	38.67 ± 1.37	< 0.001***
Apgar0 (median, IQR)	7 (2)	7.56 (1)	0.004**
Apgar5 (median, IQR)	8.43 (1)	8.89 (0)	0.006**
Birthweight (gram)	2367.39 (1280)	3322.35 (670)	< 0.001***
SBP	146.77 ± 11.90	110.58 ± 7.40	< 0.001***
DBP	88.41 ± 9.49	69.05 ± 7.66	< 0.001***

1. Data are presented as mean ± SD for normally distributed variables, and as median (IQR) for non-normally distributed variables. (*p < 0.05, **p < 0.01, ***p < 0.001, significance of effects.)

BMI, Body mass index, SBP, Systolic blood pressure, DBP, Diastolic blood pressure

**Table 2:** Dietary amino acid intakes of preeclamptic and healthy pregnant women

Variables	Preeclampsia (n = 27)	Control (n = 57)	*p* value
Alanine (mg)	2702.48 ± 171.24	3171.02 ± 53.07	< 0.001***
Arginine (mg)	3382.07 ± 555.89	4013.94 ± 239.94	< 0.001***
Aspartic Acid (mg)	5433.49 ± 646.04	6341.15 ± 390.86	< 0.001***
Phenylalanine (mg)	3353.71 ± 172.36	3374.89 ± 211.20	0.651
Glutamic acid (mg)	14,551.77 ± 2048.88	16,428.80 ± 845.65	< 0.001***
Glycine (mg)	2396.20 ± 141.73	2817.06 ± 49.12	< 0.001***
Histidine (mg)	1601.33 ± 167.19	1798.05 ± 10.17	< 0.001***
Isoleucine (mg)	3655.01 ± 583.06	3264.85 ± 41.24	0.002**
Leucine (mg)	5586.38 ± 356.24	5149.93 ± 58.72	< 0.001***
Lysine (mg)	3852.76 ± 577.00	4270.14 ± 572.86	0.003**
Methionine (mg)	1405.97 ± 148.18	1493.23 ± 125.11	0.006**
Proline (mg)	5070.99 ± 547.59	5541.96 ± 200.86	< 0.001***
Cystine (mg)	957.88 ± 219.39	1020.34 ± 78.39	0.161
Serine (mg)	3379.21 ± 476.06	3722.01 ± 274.20	0.001**
Tyrosine (mg)	2584.64 ± 106.78	2520.56 ± 216.45	0.073
Threonine (mg)	2506.01 ± 315.49	2803.25 ± 229.42	< 0.001***
Tryptophan (mg)	744.58 ± 34.42	840.99 ± 28.16	< 0.001***
Valine (mg)	3926.99 ± 483.02	3650.66 ± 43.64	0.006**
Arginine/proline ratio	0.67 ± 0.10	0.75 ± 0.12	0.007**
EAA (g)	31.73 ± 1.90	35.64 ± 0.54	< 0.001***
NEAA (g)	33.95 ± 3.25	38.07 ± 0.51	< 0.001***
BCAA (mg)	13,173.77 ± 731.37	12,047.68 ± 116.09	< 0.001***
Aromatic AA (mg)	6683.86 ± 231.00	6735.90 ± 434.30	0.476
Sulfur AA (mg)	2365.70 ± 309.65	2512.71 ± 123.03	0.024*
Dietary protein intake (g)	64.82 ± 0.30	71.84 ± 0.95	< 0.001***
Dietary animal sourced protein intake (g)	31.11 ± 0.40	28.91 ± 1.74	< 0.001***

1. Variables were shown as mean ± standard deviation (*p < 0.05, **p < 0.01, ***p < 0.00, significance of effects)

2 BCAA: Leucine, Isoleucine, Valine. EAA: Isoleucine, Valine, Leucine, Tryptophan, Phenylalanine, Methionine, Threonine, Lysine, Histidine. NEAA: Arginine, Alanine, Asparagine, Cystine, Glutamine, Glutamic Acid, Aspartic Acid, Glycine, Serine, Proline, Tyrosine. Aromatic Amino Acids: Phenylalanine, Tyrosine, Tryptophan

BCAA, Branched chain amino acids, EAA, Essential amino acids, NEAA, Non-essential amino acids

**Fig. 1:**
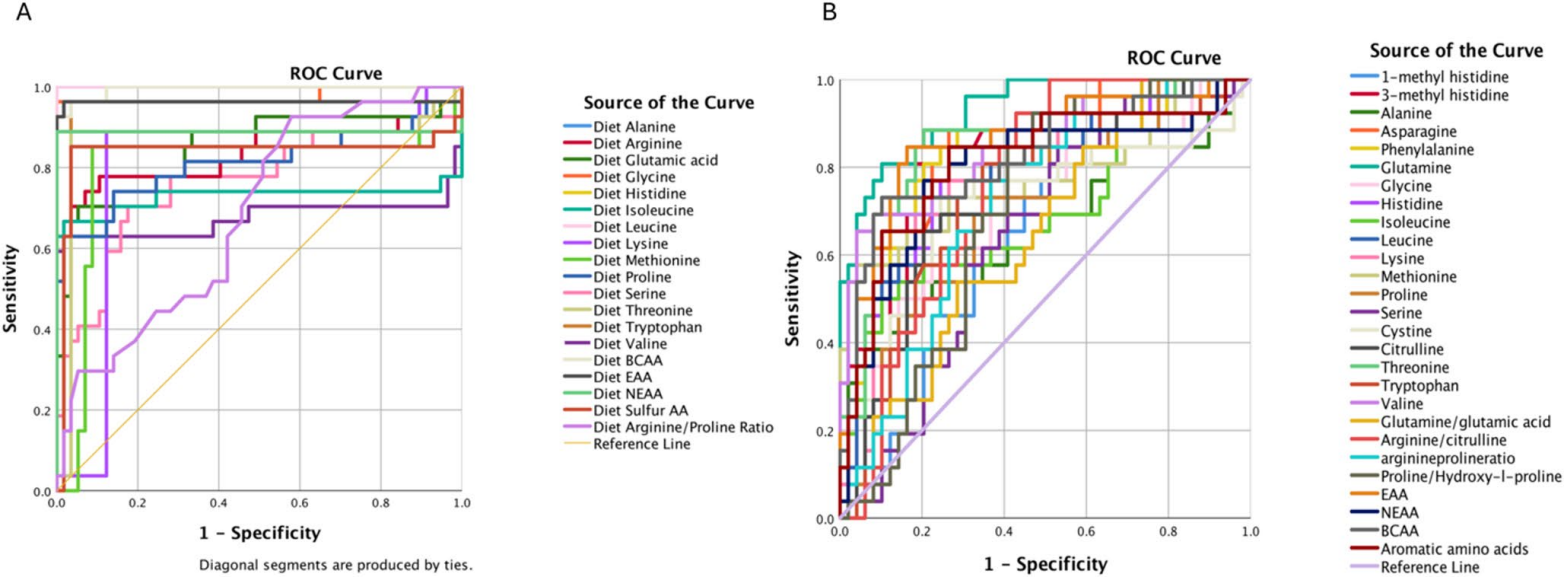
**A** ROC analysis evaluating dietary amino acid levels (adjusted for usual intake via MSM) as risk factors for preeclampsia (performed using SPSS). The ROC curves illustrate the discriminative ability of usual dietary amino acid intakes in predicting preeclampsia. Following MSM adjustment, perfect or near-perfect diagnostic performance was observed for alanine (AUC = 1.00, *p* < 0.001) and leucine (AUC = 1.00, *p* < 0.001). Additionally, BCAA (AUC = 0.99, *p* < 0.001), glycine (AUC = 0.98, *p* < 0.001), tryptophan (AUC = 0.96, *p* < 0.001), and EAA (AUC = 0.96, *p* < 0.001) exhibited very strong predictive performance. Other amino acids, including histidine, threonine, and sulfur amino acids, also showed high discriminative accuracy (AUC range: 0.84–0.89). These robust AUC values indicate that usual dietary amino acid profiles are distinctly altered in preeclampsia and may be associated with PE risk. **B** ROC analysis evaluating serum AA levels as risk factors for preeclampsia (performed using SPSS). The ROC curves illustrate the discriminative ability of serum AA levels in predicting preeclampsia. The highest AUC values were observed for glutamine (AUC = 0.93, *p* < 0.001), threonine (AUC = 0.85, *p* < 0.001), valine (AUC = 0.85, *p* < 0.001), EAA (AUC = 0.86, *p* < 0.001), and asparagine (AUC = 0.84, *p* < 0.001), indicating strong discriminative performance. Other AAs showed statistically significant but moderate predictive ability (AUC range: 0.64–0.83). These findings suggest that specific AAs may serve as potential biochemical markers associated with preeclampsia risk

ROC analysis of usual dietary amino acid intakes demonstrated that alanine (AUC = 1.00), leucine (AUC = 1.00), and total BCAAs (AUC = 0.99) exhibited excellent discriminatory value. Similarly, glycine (AUC = 0.98) and essential AAs (AUC = 0.96) showed very high diagnostic accuracy. Arginine (AUC = 0.84), histidine (AUC = 0.89), and NEAAs (AUC = 0.89) demonstrated good discrimination. In addition, valine (AUC = 0.68) and the arginine-to-proline ratio (AUC = 0.68) exhibited modest discriminatory value (Table 3) (Fig. 1A).

**Table 3:** ROC analysis to evaluate dietary amino acid levels as risk factors for preeclampsia

Variables	Cut-off	Sensitivity	Specificity	Sig	Area	Asymptotic 95% confidence interval	
						Lower bound	Upper bound
Alanine	2964.96	1	1	< 0.001	1.00	1.00	1.00
Arginine	3838.51	0.78	0.21	< 0.001	0.84	0.72	0.95
Glutamic acid	15,752.17	0.78	0.24	< 0.001	0.85	0.74	0.95
Glycine	2786.42	0.96	0.81	< 0.001	0.98	0.93	1.00
Histidine	1679.45	0.89	1	< 0.001	0.89	0.77	1.00
Isoleucine	3294.51	0.74	0.75	0.001	0.73	0.57	0.89
Leucine	5319.33	1	1	< 0.001	1.00	1.00	1.00
Lysine	3910.95	0.89	0.88	< 0.001	0.79	0.68	0.92
Methionine	1361.32	0.85	0.90	< 0.001	0.80	0.66	0.93
Proline	5437.34	0.82	0.70	< 0.001	0.81	0.69	0.93
Serine	3549.95	0.78	0.72	< 0.001	0.76	0.64	0.89
Threonine	2578.85	0.89	0.90	< 0.001	0.87	0.75	0.98
Tryptophan	812.28	0.96	0.90	< 0.001	0.96	.89	1.00
Valine	3666.72	0.67	0.70	0.010	0.68	0.51	0.84
BCAA	12,154.49	1	0.90	< 0.001	0.99	0.98	1.00
EAA	34.78	0.96	0.90	< 0.001	0.96	0.89	1.00
NEAA	36.83	0.89	0.90	< 0.001	0.89	0.77	1.00
Sulfur AA	2379.10	0.85	0.90	< 0.001	0.84	0.70	0.97
Arginine/proline ratio	.73	0.66	0.60	0.007	0.68	0.57	0.80

^*^p < 0.05, **p < 0.01, ***p < 0.001 significance of effects

Serum levels of 1-methyl-histidine, 3-methyl-histidine, alanine, asparagine, citrulline, cysteine, glutamine, glycine, histidine, isoleucine, lysine, methionine, phenylalanine, threonine, tryptophan, and valine were significantly elevated in PE (*p* < 0.05) (Table 4).

**Table 4:** Serum amino acid levels of preeclamptic and healthy pregnant women

Variables	Preeclampsia (n = 27)	Control (n = 57)	*p* value
1-Methyl histidine (µmol/L)	5.33 ± 7.94	3.13 ± 3.37	0.029*
3- Methyl histidine (µmol/L)	2.34 ± 0.78	1.55 ± 0.44	< 0.001***
Alanine (µmol/L)	457.25 ± 122.53	391.87 ± 76.19	0.023*
Alfa amino butyric acid (µmol/L)	15.92 ± 10.95	12.53 ± 6.82	0.261
Arginine (µmol/L)	201.87 ± 44.07	222.4 ± 70.08	0.235
Asparagine (µmol/L)	55.6 ± 11.35	40.67 ± 9.89	< 0.001***
Aspartic acid (µmol/L)	47.09 ± 15.13	41.38 ± 16.93	0.077
Citrulline (µmol/L)	25.4 ± 6.29	20.29 ± 5.81	< 0.001***
Cystine (µmol/L)	3.17 ± 2.42	2.23 ± 0.36	0.002**
Glutamic acid (µmol/L)	131.29 ± 45.41	127.5 ± 61.9	0.292
Glutamine (µmol/L)	464.72 ± 68.29	327.42 ± 75.6	< 0.001***
Glycine (µmol/L)	262.72 ± 48.8	228.56 ± 52.91	0.004**
Histidine (µmol/L)	117.02 ± 36.97	91.51 ± 17.36	< 0.001***
Hydroxy-l-proline (µmol/L)	10.34 ± 3.21	13.99 ± 11.83	0.292
Isoleucine (µmol/L)	75.47 ± 27.88	57.45 ± 13.87	0.002**
Leucine (µmol/L)	158.63 ± 37.79	124.55 ± 28.3	< 0.001***
Lysine (µmol/L)	193.82 ± 50.89	150 ± 40.61	< 0.001***
Methionine (µmol/L)	29.33 ± 7.90	21.82 ± 4.23	< 0.001***
Ornithine (µmol/L)	47.76 ± 20.3	48.55 ± 30.1	0.672
Phenylalanine (µmol/L)	127.67 ± 28.89	95.28 ± 21.99	< 0.001***
Proline (µmol/L)	217.10 ± 54.17	179.03 ± 50.42	0.003**
Serine (µmol/L)	179.74 ± 32.32	165.49 ± 38.88	0.055
Taurin (µmol/L)	77.99 ± 28.67	77.83 ± 27.99	0.962
Threonine (µmol/L)	260.17 ± 104.2	167.68 ± 45.55	< 0.001***
Tryptophan (µmol/L)	43.82 ± 8.81	37.37 ± 9.73	0.002**
Tyrosine (µmol/L)	62.29 ± 13.1	51.06 ± 8.23	< 0.001***
Valine (µmol/L)	233.02 ± 56.29	163.61 ± 31.29	< 0.001***
Arginine/Citrulline	8.23 ± 2.08	11.73 ± 4.72	< 0.001***
Arginine/Ornithine	5.07 ± 2.58	6.54 ± 4.35	0.203
Arginine/Proline	0.98 ± 0.32	1.33 ± 0.51	0.003**
Citrulline/Ornithin	0.62 ± 0.26	0.54 ± 0.29	0.178
Glutamine/Glutamic acid	3.96 ± 1.49	3.15 ± 1.52	0.031*
Ornithine/Proline	0.22 ± 0.84	0.27 ± 0.13	0.261
Proline/OHProline	22.21 ± 6.44	17.4 ± 9.71	0.011*
BCAA (µmol/L)	467.12 ± 113.1	345.57 ± 67.65	< 0.001***
EAA (µmol/L)	1238.94 ± 267.09	907.852 ± 173.17	< 0.001***
NEAA (µmol/L)	2082.86 ± 312.13	1774.70 ± 254.82	< 0.001***
Aromatic amino acids (µmol/L)	233.78 ± 47.43	183.72 ± 33.52	< 0.001***
Sulphur amino acids (µmol/L)	110.49 ± 30.91	102.21 ± 30.08	0.245

1. Variables were shown as mean ± standard deviation (*p < 0.05, **p < 0.01, ***p < 0.00, significance of effects)

2 BCAA: Leucine, Isoleucine, Valine. EAA: Isoleucine, Valine, Leucine, Tryptophan, Phenylalanine, Methionine, Threonine, Lysine, Histidine. NEAA: Arginine, Alanine, Asparagine, Cystine, Glutamine, Glutamic Acid, Aspartic Acid, Glycine, Serine, Proline, Tyrosine. Aromatic Amino Acids: Phenylalanine, Tyrosine, Tryptophan

BCAA, Branched chain amino acids, EAA, Essential amino acids, NEAA, Non-essential amino acids

**Fig. 2:**
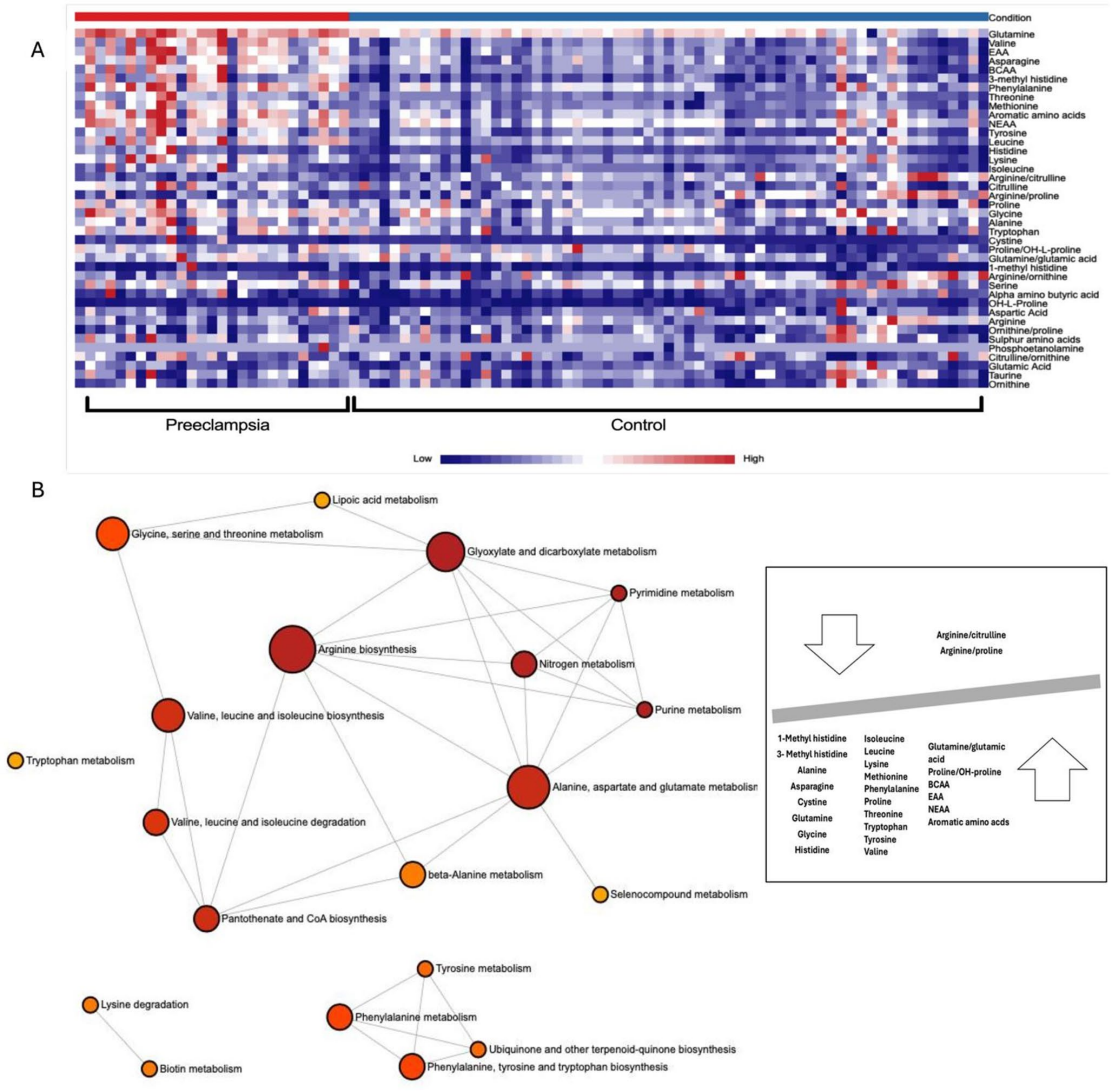
**A** Heatmap serum AAs significantly altered in preeclampsia. The heatmap highlights changes mainly in glutamine, valine, EAA, asparagine, BCAA, and 3-methyl histidine. Color intensity represents relative metabolite abundance, with darker shades indicating higher or lower levels depending on the color. The analysis indicates distinct clustering and increased levels of these metabolites in the preeclampsia group. **B** Metabolic pathway analysis of serum AAs in preeclampsia using MetaboAnalyst. The figure illustrates significantly altered metabolic pathways associated with preeclampsia based on serum AA profiles. The most impacted pathways included glyoxylate and dicarboxylate metabolism, arginine biosynthesis, alanine, aspartate and glutamate metabolism, valine, leucine and isoleucine biosynthesis, and glycine, serine and threonine metabolism. Pathway analysis was performed using MetaboAnalyst 6.0

Furthermore, significant alterations in specific serum AA ratios were observed. The PE group exhibited lower arginine/citrulline (*p* < 0.001) and arginine/proline (*p* < 0.01) ratios. Conversely, the ratios for glutamine/glutamic acid (*p* = 0.03) and proline/OH-proline (*p* = 0.01), along with the concentrations of BCAA (*p* < 0.001), essential (EAA) (*p* < 0.001) and NEAA (*p* < 0.001) and total aromatic AA (*p* < 0.001), were all significantly higher in the PE (Table 4). The heatmap is illustrated in Fig. 2A.

In the ROC analysis, serum glutamine demonstrated excellent discriminatory value (AUC = 0.93). Several other serum AAs exhibited very good discriminatory values (AUC 0.80–0.90), including 3-methylhistidine, asparagine, phenylalanine, threonine, and valine. Furthermore, the subgroup analysis showed that serum EAA (AUC = 0.859), total BCAA (AUC = 0.83), and total aromatic AA (AUC = 0.812) also showed high discriminatory value. NEAA (AUC = 0.791) were identified as risk factors associated with the condition (Table 5) (Fig. 1B). Metabolic pathway analysis is shown in Table 6 and illustrated in Fig. 2B.

**Table 5:** ROC analysis to evaluate serum amino acid levels as risk factors for preeclampsia

Variables	Cut-off	Sensitivity	Specificity	Sig	Area	Asymptotic 95% confidence interval	
						Lower bound	Upper bound
1-Methyl histidine	1.89	0.73	0.55	0.020*	0.664	0.541	0.787
3- Methyl histidine	1.58	0.85	0.83	< 0.001***	0.825	0.723	0.928
Alanine	390.44	0.65	0.53	0.027*	0.656	0.515	0.798
Asparagine	47.72	0.81	0.78	< 0.001***	0.838	0.746	0.929
Phenylalanine	106.05	0.81	0.78	< 0.001***	0.818	0.707	0.928
Glutamine	381.83	0.85	0.78	< 0.001***	0.930	0.875	0.985
Glycine	245.69	0.73	0.64	0.004**	0.705	0.579	0.831
Histidine	97.64	0.77	0.66	< 0.001***	0.785	0.674	0.896
Isoleucine	59.27	0.65	0.54	0.004**	0.704	0.575	0.833
Leucine	125.89	0.77	0.64	< 0.001***	0.783	0.670	0.895
Lysine	160.94	0.77	74	< 0.001***	0.782	0.670	0.894
Methionine	24.3	0.73	0.72	< 0.001***	0.774	0.649	0.899
Proline	185.36	0.73	0.66	0.003**	0.709	0.587	0.830
Serine	170.1	0.66	0.62	0.042*	0.644	0.518	0.769
Cysteine	2.28	0.77	0.68	0.002**	0.719	0.584	0.854
Citrulline	19.49	0.85	0.64	< 0.001***	0.752	0.638	0.866
Threonine	192.1	0.88	0.80	< 0.001***	0.853	0.757	0.949
Tryptophan	37.18	0.81	0.66	0.001**	0.732	0.611	0.852
Valine	176.12	0.77	0.66	< 0.001***	0.850	0.755	0.945
Arginine/citrulline	0.09	0.77	0.64	< 0.001***	0.759	0.653	0.865
Arginine/proline	0.88	0.77	0.60	0.002**	0.721	0.607	0.836
Glutamine/glutamic acid	3.26	0.66	0.54	0.041*	0.644	0.519	0.770
Proline/Hydroxy-l-proline	16.46	0.73	0.60	0.004**	0.701	0.584	0.817
BCAA	360.01	0.77	0.70	< 0.001***	827	0.723	0.932
EAA	991.97	0.85	0.78	< 0.001***	0.859	0.765	0.954
NEAA	1881.41	0.81	0.74	< 0.001***	0.791	0.673	0.910
Aromatic amino acids	196.92	0.84	0.75	< 0.001***	0.812	0.701	0.924

^*^p < 0.05, **p < 0.01, ***p < 0.001: indicates significance of effects

**Table 6:** Over-representation analysis of different metabolites in preeclampsia vs controls based on the Kyoto Encyclopedia of Genes and Genome (KEGG)

Pathway name	Match status	*p* value	− log(p)	Holm p	Impact
Glyoxylate and dicarboxylate metabolism	4/32	3.09 × 10⁻^12^	11.509	9.91 × 10⁻^11^	0.11
Purine metabolism	1/70	4.63 × 10⁻^12^	11.335	1.43 × 10⁻^10^	0.00
Pyrimidine metabolism	1/39	4.63 × 10⁻^12^	11.335	1.43 × 10⁻^10^	0.00
Nitrogen metabolism	2/6	9.13 × 10⁻^12^	11.04	2.65 × 10⁻^1^	0.00
Arginine biosynthesis	6/14	9.43 × 10⁻^12^	11.026	2.65 × 10⁻^10^	0.505
Alanine, aspartate and glutamate metabolism	5/28	1.17 × 10⁻^10^	9.9301	3.17 × 10⁻^9^	0.534
Pantothenate and CoA biosynthesis	2/20	2.26 × 10⁻^9^	9.6456	5.88 × 10⁻^9^	0.00
Valine, leucine and isoleucine biosynthesis	3/8	4.02 × 10⁻^9^	9.3959	1.00 × 10⁻^8^	0.00
Valine, leucine and isoleucine degradation	2/40	1.44 × 10⁻^9^	8.8404	3.47 × 10⁻^8^	0.00
Phenylalanine, tyrosine and tryptophan biosynthesis	2/4	1.07 × 10⁻^7^	6.9714	2.46 × 10⁻^7^	1.00
Phenylalanine metabolism	2/8	1.07 × 10⁻^7^	6.9714	2.46 × 10⁻^7^	0.357
Glycine, serine and threonine metabolism	3/33	2.19 × 10⁻^6^	6.6585	4.61 × 10⁻^6^	0.474
Tyrosine metabolism	1/42	6.41 × 10⁻^6^	5.1934	1.28 × 10⁻^4^	0.139
Ubiquinone and other terpenoid-quinone biosynthesis	1/19	6.41 × 10⁻⁶	5.1934	1.28 × 10⁻^4^	0.00
Beta-Alanine metabolism	2/21	3.71 × 10⁻^5^	4.4301	6.69 × 10⁻^4^	0.00

In the comparison of maternal serum amino acid profiles between early-onset (< 34 weeks) and late-onset (≥ 34 weeks) preeclampsia, serum tryptophan levels were significantly lower in the early-onset group (37.71 ± 6.40) compared to the late-onset (46.02 ± 9.42; p = 0.05), while no other serum amino acids showed statistical significance. Regarding dietary habits, the late-onset group had significantly higher daily dietary intakes of leucine (*p* = 0.04), isoleucine (*p* = 0.04), and methionine (*p* = 0.04). Consistent with these findings, the total dietary intake of BCAA was significantly elevated in the late-onset group (13,324.87 ± 776.10 mg/day) compared to the early-onset group (12,742.03 ± 342.00 mg/day; *p* = 0.01) (Online Resource 1).

Multivariable logistic regression analysis was conducted to identify independent predictors of preeclampsia (Table 7). The model demonstrated an excellent fit (Hosmer–Lemeshow *p* = 0.999) and accurately classified 92.2% of the cases. Total dietary protein intake (aOR: 0.720, 95% CI 0.564–0.921, *p* = 0.009), multiparity (aOR: 0.164, 95% CI 0.037–0.723, *p* = 0.017) and later gestational age at delivery (aOR: 0.223, 95% CI 0.073—0.683, *p* = 0.009) were confirmed as independent factors. Conversely, higher animal protein intake (aOR: 1.229, 95% CI 1.004–1.503, *p* = 0.045), increased maternal age (aOR: 1.362, 95% CI 1.070–1.734, *p* = 0.012), and higher BMI (aOR: 1.718, 95% CI 1.160–2.543, *p* = 0.007) remained independently associated with an increased risk of developing preeclampsia.

**Table 7:** Multivariable logistic regression analysis of independent predictors for preeclampsia

Variables	B	SE	Wald	*p* value	aOR (95% CI)
BMI	0.541	0.200	7.298	0.007**	1.718 (1.160–2.543)
Maternal Age	0.309	0.123	6.287	0.012*	1.362 (1.070–1.734)
Parity	− 1.809	0.758	5.701	0.017**	0.164 (0.037–0.723)
Total Dietary Protein Intake	− 0.328	0.125	6.869	0.009**	0.720 (0.564–0.921)
Animal Protein Intake	0.206	0.103	4.011	0.045*	1.229 (1.004–1.503)
Gestational Age at Delivery	− 1.500	0.570	6.915	0.009**	0.223 (0.073–0.683)

^*^p < 0.05, **p < 0.01, ***p < 0.001: indicates significance of effects

## Discussion

This study reports the novel finding that specific dietary AA patterns are significantly dysregulated in PE. We aimed to provide a comprehensive examination of both dietary (measured by three 24—h dietary recalls) and serum AA profiles in preeclampsia, identifying potential AAs as risk factors. Our findings revealed that certain dietary AAs (arginine, glycine, alanine, glutamic acid, histidine, leucine, proline, threonine) and their subgroups, BCAA, sulfur AA, EAA and NEAA (AUC > 0.8), possess high discriminatory values for PE risk. This strongly suggests that dietary AA patterns play a fundamental role in the metabolic disorder underlying PE.

To the best of our knowledge, this is the first study to evaluate the dietary AA profile in PE. Although dietary AA profile is associated with hypertension, the relationship between serum levels and dietary intakes in PE has not been previously described. To date, one study has identified a significant inverse relationship between higher methionine intake and reduced risk of PE, highlighting the importance of dietary AAs [11]. Certain AAs, such as arginine, glycine, histidine, taurine, and citrulline, can directly scavenge free oxygen radicals [26]. It has been reported that BCAA increase reactive oxygen species release and proinflammatory effects in peripheral blood mononuclear cells [27].

Various hypotheses exist about the role of AAs in PE and cardiovascular diseases in the context of inflammation, endothelial dysfunction, oxidative stress, impaired protein metabolism, and changes in the placental AA transport system [28].

A key finding of this study was that dietary arginine was lower in PE and had a good discriminatory value (AUC = 0.84), indicating its relationship with endothelial function and nitric oxide synthesis in the pathophysiology of PE. Nitric oxide uses L-arginine as a precursor for synthesis [29]. Oral arginine intake has been shown to increase systemic and vascular nitric oxide (NO) synthesis in humans and other mammals by more than 25% of physiological plasma arginine concentrations [30]. Similarly, an umbrella review examining 7 meta-analyses found that oral arginine supplementation in hypertensive adults resulted in reductions in systolic and diastolic blood pressure (by 2.2–5.4 and 2.7–3.1 mmHg, respectively) [31]. Additionally, a cross-sectional study involving 2,771 participants found a positive correlation between dietary arginine intake and serum nitric oxide concentrations [32]. An increase in NO synthesis is necessary to ensure adequate maternal vascular adaptation (decreased blood pressure, vascular resistance, and increased cardiac output) during pregnancy [33]. Reduced nitric oxide activity may lead to clinical symptoms of PE, including vasoconstriction and endothelial dysfunction [34]. Although serum arginine levels were lower in PE, this difference was not statistically significant. The lack of significance may be attributed to differences in sample size.

Another important finding of this study revealed that while the PE group consumed less alanine, their serum levels were high. The high AUC value for dietary alanine (AUC = 1.00) indicates that low alanine intake might be a significant risk factor. The serum AA profile showed a disruption of "alanine, aspartate, and glutamate metabolism" in preeclamptic patients. These AAs are crucial for energy metabolism and oxidative stress responses [35]. Since alanine is a non-essential amino acid, its circulating pool is determined by dietary intake, endogenous synthesis, and protein catabolism. The elevated serum levels, despite reduced dietary intake, suggest a complex metabolic shift. Potential causes include not only increased skeletal muscle protein breakdown and decreased hepatic gluconeogenesis, but also altered endogenous synthesis. Preeclampsia is characterized by hypoxia and oxidative stress, which can upregulate glycolysis and lead to the transamination of accumulating pyruvate into alanine. Therefore, the high serum alanine is likely a combined manifestation of increased tissue release, impaired utilization, and compensatory endogenous synthesis (Table 6).

Dietary glycine intake was found to be lower in PE and showed high discriminatory value for PE risk (AUC = 0.98). According to a study conducted on 673 hypertensive Chinese individuals from the Nutritional Health Atlas project, an inverse relationship between dietary glycine levels and hypertension was found [36]. Additionally, serum glycine levels in women with PE were found to be significantly higher than those of the patients in the control group.

Another important finding was that serum and dietary BCAA were elevated in women with PE, with high discriminatory value (AUC = 0.99). Women with PE were also found to have significantly higher intakes of animal-sourced protein compared to healthy controls, which is the main dietary source of BCAA; this increase is to be expected. A prospective cohort study of 4,288 individuals revealed that increased dietary BCAA intake is associated with higher animal protein intake and a greater incidence of hypertension [12]. Since this pattern reflects common modern dietary habits, its identification as a risk factor is especially relevant to public health and a critical target for preventative strategies.

Flores-Guerrero et al. found that elevated plasma BCAA levels are linked with a higher risk of early-onset hypertension, and that this association persists even after adjusting for age, gender, body mass index, and lipid profile [37]. Cai et al. conducted a Mendelian randomization meta-analysis, which revealed that increased serum BCAA levels are associated with a statistically significant increase in the risk of hypertension [38]. This association was observed for isoleucine (OR: 1.55; 95% CI 1.25–1.93), leucine (OR: 1.55; 95% CI 1.32–1.82), and valine (OR: 1.63; 95% CI 1.39–1.91), and sensitivity analyses supported the robustness of the results.

Our findings point to the complexity of the relationship between BCAA metabolism and PE. Elevated levels of serum BCAAs, such as valine, are consistent with systemic metabolic stress and insulin resistance, which are involved in the pathogenesis of PE. It is reported that preeclamptic patients have higher serum isoleucine and valine concentrations in PE compared to healthy controls [15, 39].

Although being a risk factor for PE, BCAA are protein building blocks and strong signaling molecules that trigger the mTOR pathway, which is essential for angiogenesis, nutrient sensing, and placental development [40].

Existing research has primarily established dietary AAs as important determinants of cardiovascular health, linking them to outcomes such as arterial stiffness, hypertension, and stroke risk [9, 12, 41–43].

Our comprehensive ROC analysis identified several serum AAs with high discriminatory value for PE. Most notably, a subgroup analysis revealed that BCAA, EAA, and aromatic AA levels exhibited outstanding predictive performance (AUC > 0.8). Among all AAs, glutamine demonstrated exceptional discriminatory value (AUC > 0.9). Furthermore, 3-methylhistidine, asparagine, glutamine, phenylalanine, threonine, and valine exhibited strong discriminatory value (AUC > 0.8). These findings suggest that these metabolites may be potential risk factors in the diagnosis of PE. In a study supporting similar results, increased plasma concentrations of aromatic AA, cysteine, threonine, and glutamic acid were observed in pregnant women with PE [44].

Maternal AA levels in pre-eclamptic women and healthy controls have been compared in numerous studies. Glycine, isoleucine, phenylalanine, histidine, valine, and glutamine are the AAs most frequently altered in serum samples associated with PE [45]. Studies have reported serum histidine, tyrosine [16, 39, 46], phenylalanine [17, 46], glutamate [16, 17], isoleucine, cysteine, glutamine, and proline [39, 46] concentrations were found to be higher than in controls. "Furthermore, previous studies have reported inconsistent findings regarding the serum levels of arginine, leucine, methionine, and alanine [16, 47].

In our study, serum glutamine levels were found to be higher in pregnant women with PE, compared to healthy controls, and ROC analysis revealed that it could be an important risk factor for the development of PE (AUC = 0.93). Glutamine, the most common free AA in human serum, plays an important role in cell life and growth. Furthermore, it is known to be the main nitrogen provider for NEAA. Impaired glutamine metabolism can lead to α-ketoglutarate deficiency, thereby inhibiting the TCA cycle and arresting endothelial cell proliferation [48]. Glutamine, the most abundant free AA in human blood circulation, is important for cell survival and growth. In addition to glutamine, decreased diet glutamic acid intake is known as a risk factor for hypertension [7].

Our research indicated that serum asparagine concentrations were elevated in pregnant women with PE relative to healthy control pregnancies. Moreover, serum asparagine levels, exhibiting a good AUC value (0.83), may be regarded as a significant risk factor for the onset of PE. In muscles, L-Asp is involved in the purine-nucleotide cycle, playing a crucial role in glutamine and alanine synthesis; in these metabolic pathways, BCAAs are the primary nitrogen source for L-Asp synthesis. This suggests that there may be a problem with amino acid metabolism and/or celullular uptake and usage [49].

Asparagine has been noted to improve endothelial dysfunction resulting from disrupted glutamine metabolism within endothelial cells. When glutamine catabolism is compromised, endothelial cells may utilize alternative nitrogen sources [50].

In our study, serum citrulline levels were found to be higher in the PE group compared to controls. Conversely, no significant difference was observed between the PE and control groups in terms of serum arginine levels. Our findings are consistent with the study conducted by Karakus et al. [51]. We hypothesize that pathways in the urea cycle are important in the development of PE. Elevated serum citrulline levels may be connected with decreased argininosuccinate synthase (ASS) or argininosuccinate lyase (ASL) enzyme. Elevated serum asparagine levels indicate active aspartate metabolism. However, aspartate or asparagine can accumulate if this pathway fails to reach arginine. Serum ornithine levels do not significantly drop, which suggests that the urea cycle's initial steps are working properly but that there might be a flaw in the later stages.

In our study, serum phenylalanine, tyrosine, tryptophan, and total aromatic AA levels were found to be significantly higher in pregnant women with PE compared to healthy controls. This finding is consistent with previous reports by Lopez-Quesada et al. and Prameswari et al. who also reported elevated maternal serum phenylalanine and tyrosine levels in PE [17, 46]. Although some studies observed similar trends without reaching statistical significance [15, 17, 39] our data supports the accumulation of aromatic AAs in the maternal circulation.

Phenylalanine may inhibit the production of tetrahydrobiopterin (BH4), a cofactor for the hydroxylation of aromatic AA involved in vasodilation. In situations where aromatic AA are present in high concentrations, BH4 oxidation may cause changes in the endothelium [52]. Consequently, the depletion of BH4 due to high phenylalanine levels may impair nitric oxide synthesis, leading to vasoconstriction.

Furthermore, Liu et al. has suggested that the elevated tyrosine levels may alter nitrogen bioavailability by affecting angiogenesis pathways in the maternal placenta [16]. Finally, regarding tryptophan, this amino acid is a precursor to serotonin, a monoaminergic neurotransmitter. Serotonin receptors are found in adrenergic nerves at the sympathetic-vascular junction, which explains their role in vascular tone [53]. Therefore, the simultaneous elevation of these three aromatic amino acids suggests a multi-pathway disruption contributing to the hypertensive phenotype of preeclampsia.

In our study, serum cysteine levels were elevated in women with PE. Cysteine is synthesized from homocysteine and can regulate the activities of several enzymes, such as vascular endothelial nitric oxide (NO) synthase (eNOS), by forming transient catalytic intermediates as part of their active sites [54]. A meta-analysis by Fan et al. found that for every 5 μmol/L increase in total homocysteine levels, the risk of hypertension increased by 1.32-fold [55].

The lack of a significant difference in dietary cysteine levels between the PE and control groups in our study may be related to sample size. When cysteine supply is low in metabolism, extracellular glutathione degradation contributes to cysteine release to maintain adequate intracellular concentrations. Therefore, glutathione acts as a cysteine reservoir [56]. In conditions where oxidative stress increases, such as PE, it was thought that low cysteine intake could contribute to the exacerbation of oxidative stress, as cysteine is known to be a limiting factor in glutathione synthesis [57].

Our study revealed a significantly lower intake of methionine in the preeclampsia group compared to controls, consistent with the recent work of Ma et al. who demonstrated that a higher dietary intake of one-carbon metabolism-related nutrients, including methionine, is associated with a significantly lower risk of preeclampsia [11]. They suggested that adequate intake of these nutrients is crucial for optimal placental development and DNA methylation. Interestingly, despite this lower dietary intake, we observed elevated serum methionine levels in pregnant women with PE compared to the control group. This contradiction between low intake and high circulating levels may suggest a metabolic disruption in methionine metabolism or transport. In preeclampsia, the maternal body may fail to convert methionine into downstream metabolites (such as SAM or cysteine) or transport it across the placenta, leading to its accumulation in the maternal circulation. Elevated homocysteine, a by-product formed during methionine metabolism, can impair endothelial function by increasing the production of asymmetric dimethylarginine (ADMA), and thus inhibiting nitric oxide synthesis [58]. Studies on methionine supplementation in both animals and humans have suggested that it indirectly affects blood pressure by raising homocysteine levels [59, 60].

The present study has several limitations that should be acknowledged. The present study has several limitations that should be acknowledged. To overcome the limitation of day-to-day variability inherent in this method, we applied the Multiple Source Method (MSM) to estimate the 'usual intake' of participants, thereby providing a more accurate reflection of their long-term nutritional habits. Also, the absence of placental tissue analysis may have limited our ability to understand placental metabolism and uptake. Nevertheless, the interdisciplinary approach—evaluating both dietary intake and serum levels simultaneously—is a significant strength of this study, offering novel insights into the metabolic alterations and 'functional deficiencies' observed in preeclampsia.

## Conclusions

The combined analysis of serum and dietary AA profiles in PE has been performed for the first time in the literature in this study. Changes in dietary arginine, alanine, glycine, glutamic acid, histidine, leucine, proline, threonine, BCAA, sulfur amino acids, EAA and NEAA (AUC > 0.8) intakes were observed. Glutamine, asparagine, phenylalanine, 3-methylhistidine, cysteine, threonine, valine, BCAA, EAA, and aromatic AA (AUC > 0.8) in serum were found to be potential risk factors for PE.

The results indicate that serum and dietary AAs may lead to endothelial dysfunction, impair vascular smooth muscle function, or have proinflammatory effects through different biochemical pathways. As a result, imbalances in AA metabolism in PE are thought to be associated with oxidative stress, endothelial damage, placental ischemia, and maternal systemic inflammation.

The findings of this study indicate that diet-derived AA metabolism in PE is a risk factor. Nutritional monitoring during pregnancy, and perhaps even before, is important for reducing the risk of PE. Longitudinal and multi-omic integrated approaches are needed to fully elucidate the underlying mechanisms. Future studies may pave the way for approaches to reduce the risk of PE through multidisciplinary strategies.

## Supplementary Information

Below is the link to the electronic supplementary material.

Supplementary Material 1

## Data Availability

The datasets generated and analyzed during the current study are not publicly available due to ethical and legal regulations in Turkey, but are available from the corresponding author on reasonable request.
