# Supplementary material for: Amino acids: Missing link in preeclampsia pathogenesis?
Source: Eur J Nutr. 2026 May 14;65(4):132. doi: 10.1007/s00394-026-03977-x (PMC13176041; doi:10.1007/s00394-026-03977-x)
Supplement: Supplementary file 1 — Supplementary Material 1 [file 394_2026_3977_MOESM1_ESM.pdf]

Amino Acids: Missing Link in Preeclampsia Pathogenesis?

European Journal of Nutrition

Iclal Sena Gezer[1], Hasan Altinsoy[3], Ayse Gulcin Bastemur[3], Ozlem Dogan[4], Atakan Tanacan[3], Fatma Doga Ocal[3], Dilek Sahin[3], Nuray Yazihan[1,2*]

Correspondence: Prof. Dr. Nuray YAZIHAN

Ankara University, Faculty of Medicine, Internal Medicine, Department of Pathophysiology, Morfoloji Building, Sihhiye, Ankara, TURKEY

[*nyazihan@ankara.edu.tr](mailto:nyazihan@ankara.edu.tr)

Online Resource

Supplementary Table 1

Comparison of dietary amino acid intakes between early-onset (<34 weeks) and late-onset PE(≥34 weeks) groups.

**Variables**	**Early Onset (n=7)**	**Late Onset (n=20)**	**p-value**
**Alanine (mg)**	2723.22 ± 11.10	2695.22 ± 199.69	0.72
**Arginine (mg)**	3122.70 ± 288.71	3472.85 ± 602.56	0.16
**Aspartic Acid (mg)**	5223.71 ± 226.28	5506.91 ± 730.13	0.33
**Phenylalanine (mg)**	3354.66 ± 231.47	3353.38 ± 154.05	0.99
**Glutamic Acid (mg)**	14190.05 ± 1879.07	14678.37 ± 2136.45	0.60
**Glycine (mg)**	2381.87 ± 9.96	2401.22 ± 165.40	0.76
**Histidine (mg)**	1578.86 ± 8.10	1609.19 ± 194.88	0.69
**Isoleucine (mg)**	3276.37 ± 391.92	3787.54 ± 587.70	0.04*
**Leucine (mg)**	5440.73 ± 15.21	5637.36 ± 403.78	0.04*
**Lysine (mg)**	3785.01 ± 24.66	3876.47 ± 673.14	0.73
**Methionine (mg)**	1344.11 ± 8.42	1427.62 ± 167.69	0.04*
**Proline (mg)**	5022.26 ± 517.10	5088.05 ± 569.83	0.79
**Cystine (mg)**	871.86 ± 109.96	987.99 ± 241.59	0.24
**Serine (mg)**	3171.43 ± 359.00	3451.93 ± 497.95	0.19
**Tyrosine (mg)**	2551.39 ± 47.46	2596.27 ± 119.75	0.35

**Threonine (mg)**	2449.36 ± 11.67	2525.83 ± 366.83	0.59
**Tryptophan (mg)**	732.73 ± 3.58	748.72 ± 39.34	0.30
**Valine (mg)**	3842.87 ± 377.33	3956.43 ± 520.37	0.60
**EAA (g)**	31.51 ± 0.16	31.81 ± 2.22	0.72
**NEAA (g)**	33.20 ± 0.20	34.22 ± 3.76	0.48
**Total BCAA (mg)**	12742.03 ± 342.00	13324.87 ± 776.10	0.01*
**Aromatic AA (mg)**	6632.76 ± 156.45	6701.75 ± 252.97	0.51
**Sulfur AA (mg)**	2249.51 ± 12.59	2406.36 ± 352.76	0.06
**Arginine / Proline Ratio**	0.62 ± 0.07	0.69 ± 0.10	0.10
**Dietary protein intake (g)**	64.69 ± 0.35	64.87 ± 0.27	0.18
**Diet Animal Protein (g)**	31.03 ± 0.42	31.14 ± 0.39	0.54

1 Variables were shown as mean ± standard deviation (*$p < 0.05$, **$p < 0.01$, ***$p<0.00$, significance of effects).
2 BCAA: Leucine, Isoleucine, Valine. EAA: Isoleucine, Valine, Leucine, Tryptophan, Phenylalanine, Methionine, Threonine, Lysine, Histidine. NEAA: Arginine, Alanine, Asparagine, Cystine, Glutamine, Glutamic Acid, Aspartic Acid, Glycine, Serine, Proline, Tyrosine. Aromatic Amino Acids: Phenylalanine, Tyrosine, Tryptophan.
BCAA, Branched chain amino acids, EAA, Essential amino acids, NEAA, Non-essential amino acids.

Supplementary Table 2

Comparison of maternal serum amino acid levels between early-onset (<34 weeks) and late-onset PE (≥34 weeks) groups

Variables	Early-Onset (n=7)	Late-Onset (n=20)	p-value*
**1-Methyl Histidine (µmol/L)**	3.81 ± 4.13	5.90 ± 9.78	0.64
**3-Methyl Histidine (µmol/L)**	2.28 ± 0.99	2.29 ± 0.76	0.65
**Alanine (µmol/L)**	446.34 ± 183.00	464.73 ± 103.79	0.76
**Alfa-Amino-n-butyric acid (µmol/L)**	18.14 ± 13.18	15.74 ± 11.51	0.51
**Arginine (µmol/L)**	180.66 ± 40.90	219.60 ± 43.59	0.06
**Asparagine (µmol/L)**	52.04 ± 12.86	57.69 ± 11.79	0.32
**Aspartic Acid (µmol/L)**	40.02 ± 12.49	44.56 ± 10.32	0.37
**Citrulline (µmol/L)**	23.50 ± 5.91	27.20 ± 6.67	0.22
**Cystine (µmol/L)**	4.74 ± 4.36	2.79 ± 0.63	0.28
**Glutamic Acid (µmol/L)**	109.86 ± 45.84	127.91 ± 39.97	0.35
**Glutamine (µmol/L)**	479.68 ± 71.88	454.92 ± 74.51	0.47
**Glycine (µmol/L)**	254.78 ± 54.25	262.82 ± 53.66	0.74
**Histidine (µmol/L)**	125.70 ± 65.59	113.50 ± 19.71	0.64
**Hydroxy-L-Proline (µmol/L)**	10.29 ± 3.67	11.18 ± 3.02	0.55

**Isoleucine (µmol/L)**	82.17 ± 36.02	79.01 ± 24.81	0.81
**Leucine (µmol/L)**	152.88 ± 50.83	156.08 ± 36.06	0.86
**Lysine (µmol/L)**	178.91 ± 69.10	205.40 ± 46.46	0.29
**Methionine (µmol/L)**	29.03 ± 6.96	31.63 ± 7.96	0.46
**Ornithine (µmol/L)**	45.62 ± 26.10	48.63 ± 20.67	0.77
**Phenylalanine (µmol/L)**	117.81 ± 33.33	129.98 ± 29.45	0.39
**Proline (µmol/L)**	195.76 ± 60.43	229.49 ± 54.92	0.20
**Serine (µmol/L)**	161.85 ± 18.74	178.63 ± 31.47	0.20
**Taurine (µmol/L)**	75.70 ± 43.21	78.52 ± 25.50	0.84
**Threonine (µmol/L)**	282.30 ± 161.32	250.96 ± 80.79	0.54
**Tryptophan (µmol/L)**	37.71 ± 6.40	46.02 ± 9.42	0.04*
**Tyrosine (µmol/L)**	57.85 ± 14.19	64.65 ± 13.39	0.28
**Valine (µmol/L)**	229.95 ± 76.48	239.12 ± 53.83	0.74
**BCAA (µmol/L)**	464.99 ± 159.01	474.21 ± 107.99	0.87
**EAA (µmol/L)**	1236.44 ± 380.77	1251.70 ± 243.50	0.92
**NEAA (µmol/L)**	1983.58 ± 390.04	2107.79 ± 304.73	0.42
**Aromatic AA (µmol/L)**	213.37 ± 53.09	240.65 ± 47.80	0.24
**Sulfur AA (µmol/L)**	109.46 ± 45.37	112.93 ± 27.98	0.82
**Arginine / Ornithine**	5.66 ± 4.12	5.17 ± 1.95	0.77
**Arginine / Citrulline**	8.15 ± 2.84	8.35 ± 1.95	0.84
**Glutamine / Glutamic acid**	5.06 ± 2.10	3.79 ± 0.97	0.06
**Arginine / Proline**	1.02 ± 0.41	1.01 ± 0.32	0.95
**Ornithine / Proline**	0.23 ± 0.10	0.22 ± 0.09	0.78
**Proline / OH-Proline**	20.79 ± 7.73	21.12 ± 5.04	0.90

1 Variables were shown as mean ± standard deviation (*$p < 0.05$, **$p < 0.01$, ***$p<0.00$, significance of effects).
2 BCAA: Leucine, Isoleucine, Valine. EAA: Isoleucine, Valine, Leucine, Tryptophan, Phenylalanine, Methionine, Threonine, Lysine, Histidine. NEAA: Arginine, Alanine, Asparagine, Cystine, Glutamine, Glutamic Acid, Aspartic Acid, Glycine, Serine, Proline, Tyrosine. Aromatic Amino Acids: Phenylalanine, Tyrosine, Tryptophan.
BCAA, Branched chain amino acids, EAA, Essential amino acids, NEAA, Non-essential amino acids.
